# Tumor size measured by multidetector CT in resectable colon cancer: correlation with regional lymph node metastasis and N stage

**DOI:** 10.1186/s12957-021-02292-5

**Published:** 2021-06-16

**Authors:** Anna Mou, Hang Li, Xiao-li Chen, Yang-hua Fan, Hong Pu

**Affiliations:** 1 Department of Radiology, Sichuan Academy of Medical Sciences and Sichuan Provincial People’s Hospital, 32# Second Section of First Ring Rd, Qingyang District, Chengdu, 610072 China; 2 Chinese Academy of Sciences Sichuan Translational Medicine Research Hospital, Chengdu, 610072 China; 3 Department of Radiology, Sichuan Cancer Hospital, Chengdu, 610072 China; 4 Department of Neurosurgery, Peking Union Medical College Hospital, Chinese Academy of Medical Sciences and Peking Union Medical College, Beijing, 100032 China

**Keywords:** Colon cancer, Lymph node metastasis, Enhanced CT, Tumor size, N stage

## Abstract

**Background:**

Lymph node metastasis (LNM) is a risk factor for poor long-term outcomes and a prognostic factor for disease-free survival in colon cancer. Preoperative lymph node status evaluation remains a challenge. The purpose of this study is to determine whether tumor size measured by multidetector computed tomography (MDCT) could be used to predict LNM and N stage in colon cancer.

**Material and methods:**

One hundred six patients with colon cancer who underwent radical surgery within 1 week of MDCT scan were enrolled. Tumor size including tumor length (Tlen), tumor maximum diameter (Tdia), tumor maximum cross-sectional area (Tare), and tumor volume (Tvol) were measured to be correlated with pathologic LNM and N stage using univariate logistic regression analysis, multivariate logistic analysis, and receiver operating characteristic (ROC) curve analysis.

**Results:**

The inter- and intraobserver reproducibility of Tlen (intraclass correlation coefficient [ICC] = 0.94, 0.95, respectively), Tdia (ICC = 0.81, 0.93, respectively), Tare (ICC = 0.97, 0.91, respectively), and Tvol (ICC = 0.99, 0.99, respectively) parameters measurement are excellent. Univariate logistic regression analysis showed that there were significant differences in Tlen, Tdia, Tare, and Tvol between positive and negative LNM (*p* < 0.001, 0.001, < 0.001, < 0.001, respectively). Multivariate logistic regression analysis revealed that Tvol was independent risk factor for predicting LNM (odds ratio, 1.082; 95% confidence interval for odds ratio, 1.039, 1.127, *p*<0.001). Tlen, Tdia, Tare, and Tvol could distinguish N0 from N1 stage (*p* < 0.001, 0.041, < 0.001, < 0.001, respectively), N0 from N2 (all *p* < 0.001), N0 from N1-2 (*p* < 0.001, 0.001, < 0.001, < 0.001, respectively), and N0-1 from N2 (*p* < 0.001, 0.001, < 0.001, < 0.001, respectively). The area under the ROC curve (AUC) was higher for Tvol than that of Tlen, Tdia, and Tare in identifying LNM (AUC = 0.83, 0.82, 0.69, 0.79), and distinguishing N0 from N1 stage (AUC = 0.79, 0.78, 0.63, 0.74), N0 from N2 stage (AUC = 0.92, 0.89, 0.80, 0.89, respectively), and N0-1 from N2 stage (AUC = 0.84, 0.79, 0.76, 0.83, respectively).

**Conclusion:**

Tumor size was correlated with regional LNM in resectable colon cancer. In particularly, Tvol showed the most potential for noninvasive preoperative prediction of regional LNM and N stage.

## Introduction

Colorectal cancer ranks third, with an estimated 1.8 million new cases, among common cancers worldwide. It is the second most common cause of cancer death, with an estimated 881,000 deaths from colorectal cancer in 2018, accounting for approximately 1/10 of cancer cases and deaths [1, 2]. Lymph node metastasis (LNM) is nearly always associated with poor long-term outcomes. Patients who have more positive lymph node involvement have lower 5-year survival rates compared to those with less lymph node involvement [3]. The 5-year overall survival rates were 83%, 76%, and 54% for patients with N0, N1, and N2 disease, respectively [4]. Moreover, according to the 7th edition of the American Joint Committee on Cancer (AJCC) staging system and the National Comprehensive Cancer Network (NCCN) Clinical Practice Guidelines for colon cancers, all patients with cT1N0M0 disease can directly receive transanal endoscopic microsurgery, while those with cT2N0M0 or cT3N0M0 disease can be recommended to undergo radical resection of colon cancer. Patients with LNM, including those with cT1-3N1M0 disease, are strongly recommended for preoperative chemoradiation therapy [5, 6]. Accurate noninvasive assessment of LNM and N stage preoperatively is crucial for effective treatment plans and predicting survival in patients with colon cancer [6].

Endorectal ultrasound and magnetic resonance imaging are widely used in the staging of rectal cancer. However, they are limited in colon cancer because of low sensitivity [7, 8]. In contrast, computed tomography (CT) is the most common method to preoperatively stage colon cancer because it can describe primary tumor shape, size, location, relationship with surrounding tissues, and the presence of distant metastasis due to its advantages of high tissue resolution, rapid scanning, and convenient follow-up [9–12]. Currently, evaluation of preoperative lymph node status based on morphological features shows poor performance, with a sensitivity of 71% and specificity of 67% because lymph node enlargement which might be caused by inflammation and microscopic metastases in small lymph nodes are difficult for radiologists to characterize [12]. The accuracy of CT for detecting LNM is 61-67% [13]. Lymph node size and shape, as a conventional method, are not reliable indicators for LNM [14, 15]. To address these issues, tumor volumetry measured on CT has been studied as a tool for predicting LNM and tumor response to therapy in rectal cancer, esophageal squamous cell carcinoma (ESCC), and gastric carcinoma [16–20]. Therefore, the purpose of this study was to determine whether tumor size measured by multidetector CT could be used to predict regional LNM and N stage in patients with colon cancer.

## Materials and methods

Ethical approval for this retrospective study was granted by the institutional review board, and the requirement for patient consent was waived because of the retrospective nature of the study.

### Patients

**Fig. 1:**
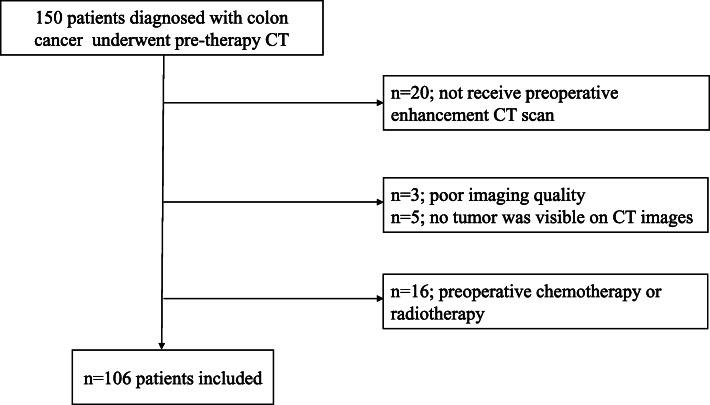
Flow diagram shows inclusion and exclusion criteria for the study

Between December 2016 and June 2018, one hundred and fifty patients diagnosed with colon cancer in our hospital were enrolled in this retrospective analysis (Fig. 1). The inclusion criteria for this study were as follows: (1) immunohistochemical testing and biopsy-proven colon cancer; (2) patient did not receive any tumor-related treatment (e.g., radiation therapy or chemotherapy) before CT; (3) tumor was considered resectable and there were no contraindications to surgery; (4) patient was subjected to contrast CT and visible colon cancer was detected on CT images. Exclusion criteria were as follows: patient did not receive preoperative enhancement CT scan (*n* = 20); the quality of the CT images was poor, or no tumor was visible on CT images (*n* = 8); preoperative chemotherapy or radiotherapy (*n* = 16). The remaining 106 patients (mean age of 62.93 years old; range from 18-83 years old) constituted the study population. According to the 7th AJCC criteria, patients were classified as having stage N0 disease if there were no metastatic lymph nodes. Stage N1a is defined as one metastatic lymph node, N1b includes two to three metastatic lymph nodes, and N1c is defined with a tumor deposited under the serosa, in the mesentery, or in the pericolonic/rectal tissues without peritoneal coverage. N2a is defined by four to six metastatic lymph nodes, and N2b includes more than seven metastatic lymph nodes [5]. Ultimately, there were 52 patients with LNM, defined as positive LNM (N+); of these 52 patients, 35 patients had N1 disease, including 13 patients with N1a, 15 with N1b, and 7 with N1c. 17 patients had N2 stage, including 11 with N2a and 6 with N2b stage. There were 54 patients without lymph node involvement, which defined as negative lymph node involvement (N0).

### CT technique

All patients underwent contrast CT on a 64-section multidetector CT system and were asked to adhere to a liquid or semiliquid diet and not to eat after eight o’clock at night the day before. Before CT image acquisition, 1000 mL of water was given orally every hour to distend the colon and increase the intestinal contrast. In order to minimize the peristaltic bowel movement, all patients received 10 mg of butylscopolamine bromide (Buscopan; Boehringer Ingelheim, Ingelheim, Germany) before the CT examination. Patients were performed in the supine position. The CT scanning variables were 120 kVp, 200–380 mA, section thickness of 8 mm, and reconstruction interval of 1-2 mm. CT scanning was performed during the arterial phase (25-30 s) and portal venous phase (60-70 s) after initiation of the contrast material injection (Ultravist 300, Iopamidol; Bayer Healthcare, Berlin, Germany) with a rate of 3 mL/s, and anatomic coverage was from the thorax to the pelvic cavity covering the entire colon.

### Tumor size parameters measurement

**Fig. 2:**
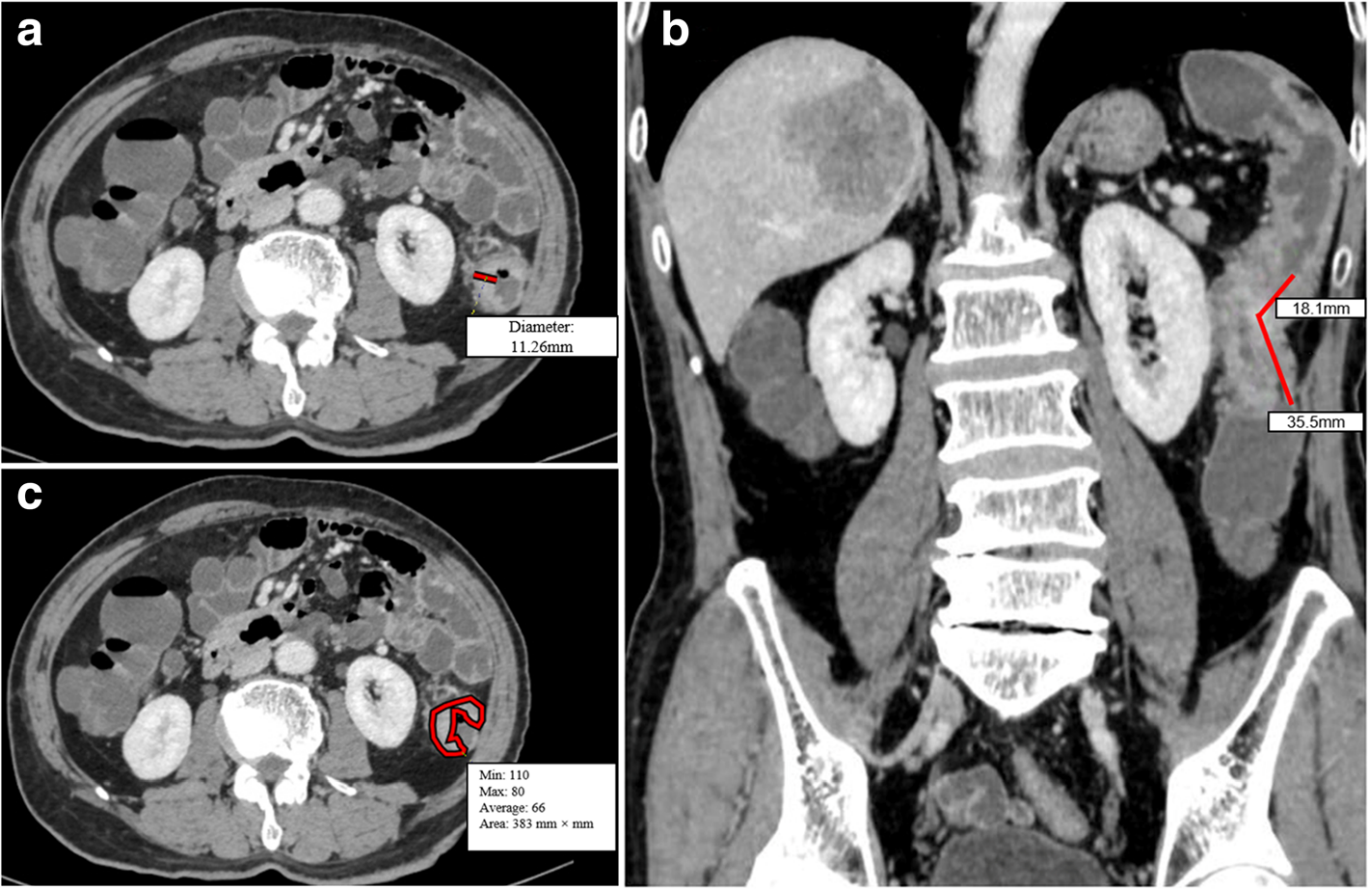
Tumor size measurement using enhanced CT in a 73-year-old man with colon cancer. **a** Tumor maximum diameter (Tdia) which is perpendicular to the colonic wall. **b** The reconstructed coronal image to measure tumor length (Tlen) with a broken line. **c** The manually drawn area along the margin of the tumor, and the value of this area (383 mm^2^) is automatically derived by software together with minimal, maximal, and average CT attenuation (in Hounsfield units)

All data were reviewed and measured on Workstation 4.4 (Advantage Workstation version 4.4; General Electric Healthcare). Coronal and sagittal views were re-established. The tumor margin was delineated by different enhancement of abnormal wall thickening and normal adjacent colon wall on the contrast CT images and corresponding noncontrast CT images. Parameters were measured on 2D images manually, as follows: (1) Tumor length (Tlen): the longest longitudinal diameter of the tumor in coronal or sagittal plane; (2) Tumor maximum diameter (Tdia): the maximum diameter of the tumor in the axial plane which is perpendicular to colonic wall; (3) Tumor maximum cross-sectional area (Tare): regions of interest (ROIs) of tumor area were drawn by tracing the lesion boundary in the axial view; (4) Tumor volume (Tvol) = sum of each axial area of ROIs of tumor × slice thickness. Pericolic lymph nodes, vessels, adjacent viscera, and lumen were carefully excluded (Fig. 2).

To estimate the accuracy of the measurement in colon cancer, patients were analyzed blindly by an experienced radiologist with 9 years of experience in abdominal radiology (H.L.) and a radiologist with 5 years of experience in radiology measurement (A.M.) for testing interobserver repeatability. The patient measurements were repeated 2 months later by a radiologist (A.M.) in order to verify the intraobserver repeatability.

### CT imaging qualitative analysis

The same two radiologists also evaluated the regional lymph node status with previous reported criteria [13, 15]. Lymph node positive was defined when the longest lymph node diameter was > 1.0 cm or < 1.0 cm in size with round shape, heterogeneity, eccentricity, hilar thinning, calcification, central necrosis, or perinodal infiltration.

### Statistical analysis

All statistical analyses were performed by SPSS software (version 22.0 for Windows; SPSS, Chicago). *P* < 0.05 was considered a significant difference. Interobserver and intraobserver measurement agreement were analyzed by calculating the intraclass correlation coefficient (ICC) (0–0.20, poor correlation; 0.21–0.40, fair correlation; 0.41–0.60, moderate correlation; 0.61–0.80, good correlation; and 0.81–1.00, excellent correlation). The relationship between tumor size and LNM were performed by Spearman rank correlation test. Clinicopathologic factors including age, sex, differentiation status, tumor location, vascular carcinoma embolus invasion, neural infiltration, and T stage were performed by univariate logistic regression analysis. Factors associated with a significant *p* value were entered in multivariate logistic regression analysis. Preoperative conventional CT-based lymph node status also was tested in multivariate logistic regression analysis. Mann-Whitney U test and receiver operating curve (ROC) characteristic analysis were performed to determine whether Tlen, Tdia, Tarea, and Tvol could predict LNM and differentiate N stage. Differences in diagnostic performance were calculated by comparing the AUCs according to the method described by DeLong et al. [21].

## Results

### Inter- and intraobserver variability of Tlen, Tdia, Tare, and Tvol measurements

The ICC of interobserver measurement for Tlen, Tdia, Tare, and Tvol is 0.94, 0.81, 0.97, and 0.99, respectively. The ICC of the intraobserver measurement by one observer is 0.95, 0.93, 0.91, and 0.99 for Tlen, Tdia, Tare, and Tvol, respectively.

### Clinicopathologic factors associated with LNM

The correlation between clinicopathologic factors with LNM is shown in Table 1. Age, sex, location of colon cancer, vascular carcinoma embolus invasion, and neural infiltration exhibited no significant differences between positive and negative LNM (*p* = 0.711, 0.123, 0.415, 0.379, and 0.712, respectively). There were significant differences in degrees of differentiation, T stage, Tlen, Tdia, Tare, and Tvol between negative and positive LNM (*p* = 0.028, 0.005, < 0.001, 0.001, < 0.001, < 0.001, respectively) (Table 1). According to multivariate logistic regression analysis, Tvol was independent risk factors for predicting LNM (*p* < 0.001; odds ratio, 1.082; 95% confidence interval for odds ratio, 1.039, 1.127). In addition, the correlation between T stage and Tlen, Tdia, Tare, and Tvol is not high and correlation coefficient is less than 0.5 by Spearman test (*r* = 0.34, 0.19, 0.37, 0.36 respectively). Therefore, we thought that multicollinearity problems can be ignored when performing the multivariate logistic regression analysis.

**Table 1:** Univariate logistic regression analysis of clinicopathologic factors and tumor size associated with regional lymph node metastasis in colon cancer

Parameter	Positive lymph nodes (*N* = 52)	Negative lymph nodes (*N* = 54)	*P* value
Age (years)	62.46 ± 11.68	63.39 ± 12.93	0.711
Sex			0.123
M	24 (46.15%)	33 (61.11%)	
F	28 (53.85%)	21 (38.89%)	
Differentiation			0.028
Well	2 (3.85%)	5 (9.26%)	
Moderately	31 (59.61%)	41 (75.93%)	
Poorly	19 (36.54%)	8 (14.81%)	
Location			0.415
Left colon cancer	20 (38.46%)	25 (46.30%)	
Right colon cancer	32 (61.54%)	29 (53.70%)	
Vascular carcinoma embolus invasion			0.379
Positive	10 (19.23%)	7 (12.96%)	
Negative	42 (80.77%)	47 (87.04%)	
Neural infiltration			0.712
Positive	7 (6.38%)	6 (8.51%)	
Negative	45 (93.62%)	48 (91.49%)	
T staging			0.005
T1	0	4 (7.4%)	
T2	1 (1.92%)	10 (18.52%)	
T3	47 (90.38%)	37 (68.52%)	
T4a	4 (7.70%)	3 (5.56%)	
Tumor size			
Tlen (cm)	6.42 ± 2.17	4.25 ± 1.61	< 0.001
Tdia (cm)	2.29 ± 1.09	1.67 ± 0.76	0.001
Tare (cm^2^)	15.73 ± 6.44	8.76 ± 4.84	< 0.001
Tvol (cm^3^)	65.70 ± 56.67	23.96 ± 16.44	< 0.001

Note: *Tdia* tumor maximum diameter, *Tlen* tumor length, *Tare* tumor maximum cross-sectional area, *Tvol* tumor volume. Data are medians ± standard deviations

### Diagnostic performance for Tlen, Tdia, Tare, and Tvol in differentiation of N stage

**Fig. 3:**
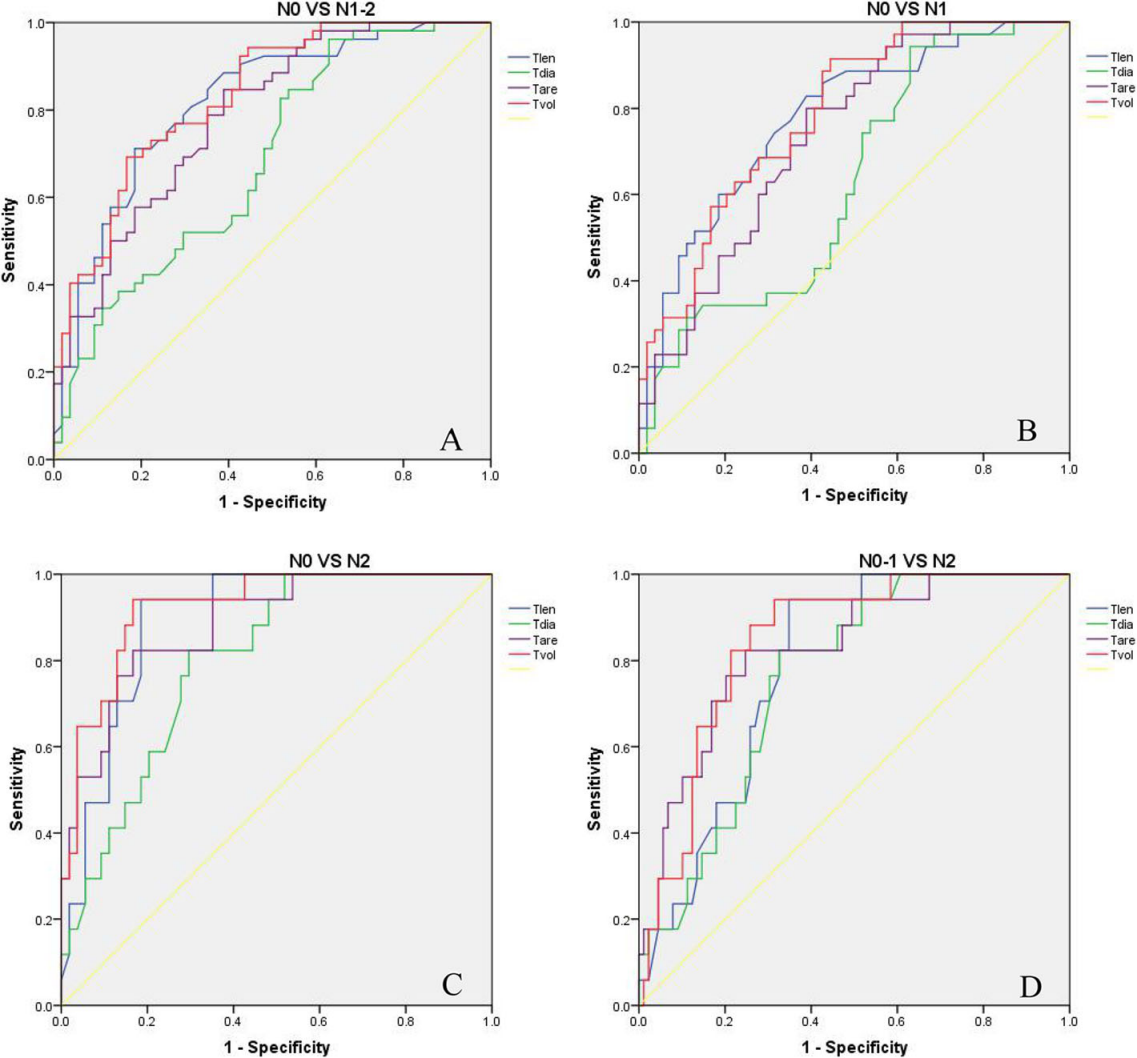
Receiver-operating curve analysis of tumor length (Tlen), tumor maximum diameter (Tdia), tumor maximum cross-sectional area (Tare), and tumor volume (Tvol) distinguishing N0 from N1-2 (**A**), N0 from N1 (**B**), N0 from N2 (**C**), N0-1 form N2 stage (**D**) in patients with colon cancer

Tlen, Tdia, Tare, and Tvol were correlated with LNM (*r* = 0.564, 0.369, 0.540, 0.610, respectively; all *p* < 0.001). There were significant differences in Tlen, Tdia, Tare, and Tvol between N0 and N1 stage (*p* < 0.001, 0.041, < 0.001, < 0.001, respectively), N0 and N2 (all *p* < 0.001), N0 and N1-2 (*p* < 0.001, 0.001, < 0.001, < 0.001, respectively), and between N0-1 and N2 (*p* < 0.001, 0.001, < 0.001, < 0.001, respectively). AUC, sensitivity, specificity, accuracy values of Tlen, Tlen, Tare, and Tvol in the differentiation of N stage in colon cancer are summarized in Table 2 and Fig. 3. From ROC analysis, the area under the ROC curve (AUC) was higher for Tvol than that of Tlen, Tdia, and Tare in identifying LNM (AUC = 0.83, 0.82, 0.69, 0.79, respectively), and distinguishing N0 from N1 stage (AUC = 0.79, 0.78, 0.63, 0.74, respectively), distinguishing N0 from N2 stage (AUC = 0.92, 0.89, 0.80, 0.89, respectively), and distinguishing N0-1 from N2 (AUC = 0.84, 0.79, 0.76, 0.83, respectively).

**Table 2:** ROC analysis of Tlen, Tdia, Tare, and Tvol in the differentiation of N stage in patients with colon cancer

Tumor size	Cutoff	AUC (95% CI)	*P* value	Sensitivity (95% CI)	Specificity (95% CI)	Accuracy (95% CI)
N0 VS N1-2						
Tlen (cm)	5.20	0.82 (0.74~0.90)	< 0.001	71.2% (57.7~81.8%, 37/52)	81.5% (69.0~90.0%, 44/54)	76.4% (67.5~83.5%, 81/106)
Tdia (cm)	1.56	0.69 (0.58~0.79)	0.001	71.2% (57.7~81.8%, 37/52)	51.9% (38.9~64.6%, 28/54)	61.3% (51.8~70.1%, 65/106)
Tare (cm^2^)	9.47	0.79 (0.70~0.87)	< 0.001	84.6% (72.2~92.3%, 44/52)	61.1% (47.8~72.3%, 33/54)	72.6% (63.4~80.3%, 77/106)
Tvol (cm^3^)	38.00	0.83 (0.74~0.90)	< 0.001	69.2% (55.7~80.2%, 36/52)	83.3% (71.0~91.2%, 45/54)	76.4% (67.5~83.5%, 81/106)
N0 VS N1						
Tlen (cm)	4.25	0.78 (0.68~0.88)	< 0.001	82.9% (66.9~92.3%, 29/35)	61.1% (47.8~73.0%, 33/54)	69.7% (59.4~78.3%, 62/89)
Tdia (cm)	1.49	0.63 (0.51~0.75)	0.041	74.3% (57.8~86.0%, 26/35)	48.1% (35.4~61.2%, 26/54)	58.4% (48.0~68.1%, 52/89)
Tare (cm^2^)	9.47	0.74 (0.64~0.84)	< 0.001	80.0% (63.8~90.2%, 28/35)	61.1% (47.8~73.0%, 33/54)	68.5% (58.3~77.3%, 61/89)
Tvol (cm^3^)	23.22	0.79 (0.70~0.88)	< 0.001	88.6% (73.5~96.1%, 31/35)	57.4% (44.2~69.7%, 31/54)	69.7% (59.4~78.3%, 62/89)
N0 VS N2						
Tlen (cm)	5.20	0.89 (0.82~0.97)	< 0.001	94.1% (71.1~100%, 16/17)	81.5% (69.0~89.8%, 44/54)	84.5% (74.2~91.3%, 60/71)
Tdia (cm)	1.99	0.80 (0.70~0.91)	< 0.001	82.4% (58.2~94.6%, 14/17)	70.4% (57.1~80.9%, 38/54)	74.6% (63.4~83.4%, 53/71)
Tare (cm^2^)	13.41	0.89 (0.80~0.97)	< 0.001	82.4% (58.2~94.6%, 14/17)	83.3% (71.0~91.2%, 45/54)	83.1% (72.3~90.2%, 59/71)
Tvol (cm^3^)	38.40	0.92 (0.86~0.99)	< 0.001	94.1% (71.1~100%, 16/17)	83.3% (71.0~91.2%, 45/54)	85.6% (75.8~92.4%, 61/71)
N0-1 VS N2						
Tlen (cm)	5.20	0.79 (0.70~0.88)	< 0.001	94.1% (71.1~100%, 16/17)	65.2% (54.8~74.3%, 58/89)	69.8% (60.5~77.8%, 74/106)
Tdia (cm)	2.00	0.76 (0.66~0.86)	0.001	82.4% (58.2~94.6%, 14/17)	67.4% (57.1~76.3%, 60/89)	69.8% (60.5~77.8%, 74/106)
Tare (cm^2^)	13.41	0.83 (0.72~0.93)	< 0.001	82.4% (58.2~94.6%, 14/17)	75.3% (65.3~85.2%, 67/89)	76.4% (67.5~83.5%, 81/106)
Tvol (cm^3^)	38.63	0.84 (0.76~0.93)	< 0.001	94.1% (71.1~100%, 16/17)	68.5% (58.3~77.3%, 61/89)	72.6% (63.4~80.3%, 77/106)

Note: *Tdia* tumor maximum diameter, *Tlen* tumor length, *Tare* tumor maximum cross-sectional area, *Tvol* tumor volume, *AUC* the area under the ROC curve, *CI* confidence interval

### Preoperative conventional CT-based lymph node status correlation with pathologic results

Preoperative conventional CT-based lymph node status correlation with pathologic results is summarized in Table 3. According to multivariate logistic regression analysis, preoperative conventional CT-based method was a risk factor for LNM (*p =* 0.027; odds ratio, 3.34; 95% confidence interval [CI] for odds ratio, 1.145, 10.035). The AUC, sensitivity, specificity, and accuracy values of the conventional CT-based method for predicting LNM were 0.726 (95% CI, 0.628-0.825), 71.2% (95% CI, 57.7~81.8%; 37/52), 74.1% (95% CI, 61.0~84.0%; 40/54), 72.6% (95% CI, 63.4~80.3%; 77/106), respectively. The AUC for Tvol was higher than that of conventional CT-based method for predicting LNM (AUC, 0.830 vs 0.726; *p =* 0.045)*.*

**Table 3:** Preoperative CT-based lymph nodes status

Preoperative CT lymph node status	Pathologic results		Total
	Positive (*n* = 52)	Negative (*n* = 54)	
Positive	37	14	51
Negative	15	40	55
Total	52	54	106

## Discussion

In this study, our preliminary results showed that tumor size measured on MDCT could be a tool for the initial prediction of LNM in colon cancer. Tvol was an independent risk factor for predicting LNM. Tlen, Tdia, Tare, and Tvol could distinguish between N0 and N1-2, N0 and N1, N0 and N2, and N0-1 and N2 stage. Tvol had the best diagnostic efficiency in identifying LNM and differentiating N stage.

Previous studies have applied different criteria based on either size and/or morphology for identifying LNM [22–24]. Another study used the radiographic criteria of lymph node diameter (> 1.0 cm) or round shape, heterogeneity, eccentricity, hilar thinning, calcification, central necrosis, and perinodal infiltration to evaluate lymph node involvement with sensitivity of 54-88%, specificity of 55-66%, accuracy of 61-70% [13]. Sibileau et al. reported the accuracy of the association of the 3 criteria (size, number, and density) with sensitivity of 77.3% and specificity of 77.4% [25]. However, metastatic lymph node smaller than 3 mm are difficult to depict with MR imaging and CT imaging and can easily be misinterpreted as small blood vessels and, most important, that some of nodes larger than 1 cm seen on CT images may be the benign. Therefore, the node seen on CT images that could not be matched with histopathologic findings were all judged to be benign on CT images. This limitation made morphological method not accurate to assess lymph node status. It is hard to ensure the metastatic lymph node we diagnosed on CT images is the one confirmed by pathology eventually.

Tumor length is an independent predictor of mortality in patients with esophageal carcinoma and a risk factor for LNM to predict survival in patients with ESCC [26, 27]. In this study, we indicated that tumor length was correlated with LNM in patients with colon cancer. This was because increasing longitudinal growth in the lymphatic-rich submucosa was a predictor of regional LNM. The longer the tumor length, the deeper the tumor invading the colonic wall and the more frequent the incidence of LNM. Tumor thickness was an independent adverse factor for LNM in oral carcinoma [28] and could be associated with LNM in ESCC [26]. In theory, the larger the tumor diameter, the deeper tumor invading the colon wall, more likely invading adjacent structures, and the higher risk of LNM. In our study, we found that Tdia was correlated with LNM, but the diagnostic efficiency of Tdia is not high in colon cancer. We thought that the inflatable dilatation of the colon and blur margin caused by tumor inflammation and invasion could affect tumor diameter measurement. For Tare, we found that Tare was also correlated with LNM. Tare is the maximum cross-sectional area of colon cancer and is used for delineating the whole circumference of the tumor. Compared with Tdia, it contains a much broader margin and could reflect tumor geometry and growth. Previous study reported the sensitivity, specificity, and accuracy of CT colonography for predicting LNM were 69.31%, 66.15%, and 67.14% respectively [29]. In this study, we found that tumor volume had the best repeatability measurement and was an independent risk factor for predicting LNM in patients with colon cancer by multivariate logistic analysis. The sensitivity, specificity, and accuracy of Tvol for predicting LNM were 69.2%, 83.3%, and 76.4%, respectively. However, some other study indicated tumor CT colonography volumetry could not predict N stage [30]. The explanation for the different results could be the relatively small sample size of LNM. First, the sample size of patients with LNM was smaller than that of our studies. Second, the sample size of patients with LNM was smaller than the sample size of patients without LNM in the published study. Usually, to be a risk factor is not equal to can be used to predict LNM. The explanation for this reason could be that most of these published studies utilized clinicopathologic factors excluding tumor size to predict LNM and concluded a risk factor is not equal to can be used to predict LNM. Quantitative parameter such as tumor volume had not been included for predicting LNM. Moreover, we demonstrated that Tvol could help differentiate N0 from N1-2, N0 from N1, N0 from N2, and N0-1 from N2 with moderately sensitivity, specificity, and accuracy. Our findings were consistent with these previous studies in other tumors. For example, Li et al. showed that tumor volume could predict regional LNM and differentiate various N stages with an accuracy of 70% in adenocarcinoma of the esophagogastric junction [20]. Tumor volume measured on MDCT could differentiate N0 and N1-3 stages, N0-1 and N2-3, and N0-2 and N3 with moderately accurate in resectable ESCC [26]. Chen et al. reported that tumor volume data had better correlation with LNM than that of the tumor length and tumor diameters [31]. We also demonstrated that Tvol had the highest AUC compared with Tlen, Tdia, and Tare in distinguishing N stage. This may be because tumor volume takes both the Tlen, Tdia, and Tare into consideration. The larger the tumor volume, the deeper the tumor invading the colonic wall and the more frequent the incidence of LNM. In addition, treatment of colon cancers in both N1 and N2 category is different. Patients with N2 category regardless of T stage should be classified into high-risk stage III. These patients with N2 colon cancer may have a poorer prognosis than those with N1 category [6]. Therefore, patients with N2 colon cancer should undergo more longer adjuvant therapy to reduce recurrence. Although our results indicated tumor size could not be used to distinguish N1 from N2 category, Tvol had good diagnostic efficacy for differentiating N0-1 from N2 with sensitivity of 94.1%, specificity of 68.5%, and accuracy of 72.6%. In our study, the AUC, sensitivity, specificity, and accuracy values of the conventional CT-based method for distinguishing N0 from N1-N2 were 0.726, 71.2%, 74.1%, and 72.6%, respectively. Tvol had a better diagnostic efficiency to evaluate LNM, with AUC, sensitivity, specificity, accuracy of 0.83, 69.2%, 83.3%, and 76.4%, respectively. The AUC for Tvol was higher than that of conventional CT-based method for predicting LNM (AUC, 0.830 vs 0.726; *p =* 0.045)*.* We can conclude that Tvol may be the best predictor to differentiate N stage in colon cancer.

Our study had several limitations. First, the collected sample number is too small and only 52 patients had LNM. Small data analysis could lead that the current result do not reflect the whole information. Lymphovascular invasion and perineural invasion have a high risk of LNM and a poor prognostic factor in colon diseases [6, 32, 33]. In our study, just few cases had positive lymphovascular or perineural invasion, which resulted in no significant differences. Second, tumor volume measurements on CT images can be time-consuming. However, in this study, the time required for well-trained radiologists to draw the whole tumor area was controlled to 10 min. In future study, semiautomatic measurement method may be used to reduce the time of measurement. Third, the measurement of Tlen, Tdia, and Tare might be affected by the distention of the contents in the intestinal canal. To minimize this effect, all patients were asked to perform bowel preparation with butylscopolamine bromide and drink 1000 mL of water before CT scanning.

In conclusion, the manual measurement of tumor length, tumor diameter, tumor area, and tumor volume on MDCT in patients with colon cancer provided good maneuverability and repeatability. Tvol had higher diagnostic efficiency in identifying LNM and differentiating N0 from N1, N0-1 from N2, and N0 from N2. Tvol could be helpful in quantitatively predicting LNM and N stage for the appropriate choice of treatment approach for this tumor.

## Data Availability

Not applicable.

## Abbreviations

MDCT: Multidetector computed tomography
LNM: Lymph node metastasis
Tlen: Tumor length
Tdia: Tumor maximum diameter
Tare: Tumor maximum cross-sectional area
Tvol: Tumor volume
ROC: Receiver operating characteristic
AUC: Area under the ROC curve
ICC: Intraclass correlation coefficient
ESCC: Esophageal squamous cell carcinoma
AJCC: American Joint Committee on Cancer
NCCN: National Comprehensive Cancer Network
CI: Confidence interval
